# Impacted Mandibular Third Molar: Approaches and Current Perspectives in Surgical Therapy

**DOI:** 10.3390/medicina61091683

**Published:** 2025-09-17

**Authors:** Federica Di Spirito, Pierpaolo Di Lorenzo, Iman Rizki, Alfonso Acerra, Francesco Giordano, Massimo Amato, Alessandro Santurro

**Affiliations:** 1 Department of Medicine, Surgery and Dentistry, University of Salerno, 84084 Salerno, Italy; i.rizki@studenti.unisa.it (I.R.); frgiordano@unisa.it (F.G.); mamato@unisa.it (M.A.); asanturro@unisa.it (A.S.); 2 Department of Advanced Biomedical Sciences, University of Naples Federico II, 80138 Naples, Italy; pierpaolo.dilorenzo@unina.it

**Keywords:** third molar, wisdom tooth, oral surgery, coronectomy, post-operative complications, nerve injury, informed consent, medico-legal issues

## Abstract

Mandibular third molar surgery is a procedure that can be accompanied by several complications, one of the most important being neurological ones, resulting from transient or permanent injury to the lingual nerve (LNI) or inferior alveolar nerve (IANI). For this reason, a conservative technique, coronectomy, has been proposed. Despite the fact that this procedure seems to have important advantages, such as IANI reduction, it turns out that it is currently a non-standardized procedure, showing several limitations and requiring a precise and well-documented informed consent process because its use may potentially increase the practitioner’s medico-legal vulnerability.

## 1. Introduction

The third molar is the dental element most frequently found to be impacted, with a frequency of occurrence ranging from 18% to 32%, and the highest rates being in Asia: up to 43.1% [1,2,3]. There are several reasons for its eruptive difficulty, such as its position within the mandible, anatomical limitations in the eruptive path, its time of eruption, the involution of the splanchnocranium in favor of the neurocranium, its embryological position in formation, and the density of the mandibular bone [4]. Due to these conditions the third molar can fail to erupt properly, a condition better known as impaired eruption [5]. However, this situation does not necessarily result in a pathological scenario. In fact, surgery of an impacted tooth is performed only if complications arise, directly or indirectly related to the condition [6]. The variability of pathological scenarios, of conditions in which the wisdom tooth can be found, has induced major scientific societies to clarify indications for intervention. For example, the National Institute for Health and Care Excellence (NICE, UK) advises against third molar avulsion for prophylactic purposes in cases of an asymptomatic, pathology-free dental element, while the American Association of Oral and Maxillofacial Surgeons (AAOMS, USA) recommends a tailor-made, case-by-case evaluation, making a risk–benefit consideration related to the specific case [7,8]. The most common complication is pericoronitis, the typical symptoms of which manifest as ear pain, difficulty swallowing, and clenching of the chewing muscles, due to antalgic contraction [9,10]. This can be exacerbated by an infection at the site of the impacted third molar, which can give far more severe complications that may even require hospitalization of the patient [11].

Complications arising from the third molar in dysodontiasis represent indications for wisdom tooth removal surgery [12]. Like any surgical treatment, third molar extraction is also potentially associated with risks related to the procedure itself [13]. The most frequently occurring complication is alveolar osteitis, which consists of clot loss within the socket and is accompanied by severe postoperative pain and halitosis. More rarely, bleeding or hemorrhage may occur, complications often related to the patient’s comorbidities or medications taken [14,15]. Neurological complications are among the most severe complications associated with lower third molar extraction, resulting from transient or permanent injury to the lingual nerve or inferior alveolar nerve [16]. Inferior alveolar nerve injury is one of the most feared complications, and may occur in about 4% of cases.

In the case of lingual nerve involvement, the injury may be stretch-related, due to lacerations or compressions [17], while in the case of inferior alveolar nerve involvement, the injury may result from direct trauma, during osteotomy or extraction, or more rarely from post-operative compression [18]. Preoperative evaluation of the inferior alveolar nerve, compared with the course of the lingual nerve, can benefit from fundamental radiological analysis. This is because the inferior alveolar nerve runs within a bony canal, which is visible radiographically and therefore gives information about its position and relationship to the wisdom tooth roots. This relationship was also classified by Maglione et al. to define the degree of difficulty of wisdom tooth extraction [19]. In fact, surgery of the impacted third molar turns out to be the leading cause of permanent injury to the inferior alveolar nerve. Kang et al. confirmed that the surgical technique used and the proximity between the tooth roots and the nerve are the determining factors for the risk of nerve damage [20]. In many cases there is a relationship of continuity between the NAI and the roots of the third molar, or these wrap around the nerve structure, with a high risk of postoperative damage. For this reason, less traumatic and less invasive approaches have been proposed that can reduce surgical trauma and promote better postoperative healing [21]. One of these is coronectomy, which aims precisely to preserve nerve structures. However, this conservative technique, described with promising results given by the preservation of anatomical structures, can cause complications too. This condition turns out to be a problem not only from a clinical point of view, since the patient could require new care, but also from a medical-legal point of view, since the procedure does not appear to be standardized to date, thus opening up to professional liability to date still unclear. The debate therefore is entirely current and relevant, and it is essential that the choice of procedure must be preceded by proper and comprehensive information and consent of the patient undergoing the surgery.

## 2. Clinical Framing and Treatment

The diagnostic, clinical, and therapeutic evaluation of the impacted third molar depends on numerous factors, starting with the patient’s medical history, current drug therapies, age, morphology of the dental element, and its relationship to the mandibular canal.

### 2.1. Epidemiology, Anatomy and Surgery of Impacted Wisdom Tooth

Impacted lower third molar is a condition that affects a significant percentage of the world’s population. Epidemiological studies report that the prevalence varies between 35.9% and 58.7% of the population, depending on race, age, and dietary habits [1].

The anatomy of the lower third molar varies greatly from individual to individual, both in terms of location, angulation, number and shape of roots, and overall tooth size [22,23]. The presence of an impacted third molar can lead to several clinical complications, including pericoronitis, an acute or chronic inflammation of the soft tissues surrounding the crown of a partially erupted tooth, which turns out to be one of the clinical indications for wisdom tooth surgery. As highlighted by Galvão et al., this condition occurs more in the vertical position of Winter and in position A of Pell and Gregory [24].

The standard procedure of wisdom tooth surgery, scientifically accepted and described by all international societies of Oral Surgery provides, following clinical and radiographic preoperative planning, to perform infiltration with local anesthetic, incision and muco-periosteal flap detachment (flap design), osteotomy to visibly expose the crown of the impacted tooth, odontotomy, these two procedures both performed through the use of a burr mounted on a turbine or micromotor under constant irrigation, successively dislocation and extraction [7,8]. In the case of divergent roots or root curvatures, root separation will also be performed prior to root avulsion. The type of osteotomy, as well as odontotomy, as well as the type of flap design will depend on the morphology, but especially on the position and depth of the dental element, taking into account the classifications of Pell and Gregory and Winter [25,26]. These classifications describe anatomically and morphologically the third molar. Specifically, they identify its position in the mesio-distal sense and depth in the apico-coronal sense. In addition, they evaluate the angulation of the wisdom tooth in three classes related to the long axis of second molar: mesio-inclined, disto-inclined, and normo-inclined. The position in the three dimensions of the third molar determines the choice of flap and avulsion surgical approach.

Avulsion will be followed by procedures to examine the alveolar cavity such as alveolar curettage, check hemostasis, apply local hemostatics if necessary, and finally suture with flap replacement.

### 2.2. Coronectomy

Coronectomy emerged in the 1980s as a conservative approach aimed at minimizing the incidence of inferior alveolar nerve injury (IANI) [27]. Initially introduced by Ecuyer and Debien in 1984, this technique has progressively gained acceptance, particularly in cases where the mandibular third molar roots are closely associated with the inferior alveolar canal, rendering full extraction more hazardous [28].

#### 2.2.1. Procedure

Coronectomy involves the deliberate removal of the crown of the mandibular third molar while retaining the roots in situ, thereby reducing the risk of direct injury to the inferior alveolar nerve. The technique includes sectioning the tooth at the cemento-enamel junction, extracting the crown, and refining the remaining root surfaces to eliminate sharp edges that may predispose to future complications.

This procedure is typically recommended when radiographic indicators point to a heightened likelihood of nerve damage—such signs include root darkening, interruption of the lamina dura, or displacement of the inferior alveolar canal. These radiographic risk factors can be reliably assessed using panoramic imaging or cone-beam computed tomography (CBCT) [29,30,31,32].

However, coronectomy is contraindicated in scenarios where the tooth is non-vital, shows periapical pathology, has extensive carious lesions, or demonstrates root mobility [33].

#### 2.2.2. Complications

A recent systematic review evaluating 8198 coronectomy procedures reported an overall complication rate of approximately 26% [34]. This procedure is typically recommended when radiographic indicators point to a heightened likelihood of nerve damage—such signs include root darkening, interruption of the lamina dura, or displacement of the inferior alveolar canal. The most prevalent outcome was root migration (Table 1), observed in around 12% of cases, although reported frequencies varied widely across studies, ranging from about 30% to nearly 80% [34,35,36,37].

This phenomenon is typically benign, as the retained roots tend to migrate away from the inferior alveolar nerve (IAN) and stabilize over time, thereby minimizing the risk of nerve injury. Bernabeu Mira et al. [38] and Póvoa et al. [39] emphasized that while root migration is a common occurrence, it seldom necessitates further intervention. In rare cases (~0.2%), however, migration can lead to root exposure, requiring delayed extraction, but it depends on follow-up period to which the patient is referred. [40].

Several studies have further characterized the dynamics of root migration. Simons et al. [41] found that most displacement occurs within the first six months postoperatively, with a mean migration of 3.3 mm at two months and 5.3 mm at six months. Younger individuals and female patients were more likely to exhibit greater migration. Hamad et al. [42] reported that root movement occurred in 74% of patients within the first postoperative year, with an average shift of 3.85 mm, while Ali et al. [43] noted that migration generally plateaued after 12 months, with stabilization by 24 months. Importantly, these studies confirm that delayed removal of migrated roots does not increase the risk of IANI, supporting the long-term safety profile of coronectomy in high-risk anatomical scenarios. Postoperative pain is another commonly reported complication (Table 2), affecting approximately 20% of patients [35,39]. Williams and Tollervey observed that, although immediate postoperative discomfort may be comparable to that of total third molar extraction, it tends to resolve more quickly following coronectomy, likely due to reduced neural trauma [44]. Nonetheless, Póvoa et al. reported higher pain levels (22%), suggesting that factors such as surgical technique, patient variability, and follow-up duration may significantly influence pain perception [39].

According Di Spirito et al. infections were reported in about 2.5% of cases, a finding consistent with the 2.4% pooled prevalence reported in the meta-analysis by Kostares et al. [34,45]. Póvoa et al. observed a slightly higher rate (3.95%) [39], while Yan et al. highlighted that infection risk can vary based on anatomical and periodontal conditions [46]. These results (Table 3) reinforce the notion that infection is an infrequent but multifactorial complication, dependent on procedural and patient-specific variables [36].

Alveolar osteitis (dry socket) occurred in approximately 1.2% of patients (Table 4), which is consistent with previous data and slightly higher than the 1.1% reported by Póvoa et al. [34,39]. However, coronectomy appears to carry a lower risk of dry socket compared to full extraction. Bernabeu-Mira et al. and Hamad et al. attributed this difference to the preservation of root structures, which may enhance bone healing [38,42]. Notably, Hamad et al. found that dry socket occurred in only 0.5% of coronectomy cases compared to 3.7% in total extractions [42].

Pulpal pathology was reported in less than 0.1% of cases [34], supporting the view that retained roots seldom become symptomatic unless exposed to the oral cavity [39].

Inferior alveolar nerve injury (IANI) was identified in 0.76% of patients [34], consistent with the 0.59% rate found by Póvoa et al. [39]. The risk of IANI is significantly reduced in coronectomy (Table 5) compared to total extraction, particularly in patients with radiographic signs of close nerve proximity [47,48].

Similarly, lingual nerve injury (LNI) was infrequent (~0.1%) [34], aligning with previous findings [39] and likely reflects the benefit of minimally invasive and atraumatic surgical approaches [49].

#### 2.2.3. Re-Intervention

Re-intervention was necessary in approximately 4.5% of cases [34], typically occurring six months or more after the initial procedure. This rate (Table 6) is consistent with values reported in the literature, though variation exists depending on clinical protocols and follow-up duration [39,50].

For instance, Póvoa et al. documented a lower reoperation rate of 1.1%, potentially reflecting differences in patient selection and surgical technique [39]. In contrast, Nowak et al. observed a rate of 3.1% [51], with most cases resulting from root migration that led to exposure or symptomatic retained roots. Their findings also indicated that younger patients and individuals with pre-existing periodontal conditions were more likely to require secondary intervention, emphasizing the role of careful case selection in coronectomy planning.

Long-term follow-up is essential for accurately assessing outcomes and identifying late complications. Ali et al. highlighted that root migration occurring beyond 12 months postoperatively was associated with an increased likelihood of reoperation [43]. Additionally, postoperative infection appears to be a significant risk factor, with Kostares et al. reporting a 3.2-fold higher chance of secondary treatment in cases complicated by surgical site infection [45]. However, re-intervention rates may be underestimated due to the lack of standardized long-term monitoring. As emphasized by Kostares et al. the implementation of consistent follow-up protocols is critical to improving the reliability of coronectomy outcome assessments [45].

Coherently, the available evidence also underscores the importance of long-term follow-up after coronectomy, as the timing of re-intervention can vary considerably. One study reported that secondary procedures were required between 6 months and 10 years postoperatively, with a mean interval of approximately 10 months [50]. Similarly, Nowak et al. observed an average re-intervention time of 12 months, with the majority (about 80%) of secondary extractions occurring within the first two years [51]. Their findings further indicated that delayed root migration—particularly beyond the 12-month mark—was the primary factor contributing to late-stage reoperation.

Bernabeu-Mira et al. also highlighted the long-term nature of potential complications, noting that root exposure and postoperative infections were the main causes of re-intervention during follow-up periods ranging from 2 to 9 years [38]. These data suggest that although the majority of retained roots remain clinically silent, a small subset may become symptomatic over time, requiring eventual extraction. Collectively, these findings reinforce the need for sustained monitoring beyond the immediate postoperative period to identify and manage delayed complications effectively.

Among the various indications for re-intervention following coronectomy, root exposure emerged as the most frequently reported, accounting for approximately 17% of all secondary procedures [14,32,34,50]. Other relatively common causes included postoperative infection (~5%), pain (~3%), and the presence of residual enamel (~3%). These findings align with those of Agbaje et al. [37], who also identified root exposure as a primary complication requiring delayed extraction. Their analysis further pointed to periapical infection and persistent mobility of the retained root segments as significant predictors of re-intervention. Similarly, Nowak et al. emphasized that while root migration is a commonly observed sequela of coronectomy, it is generally asymptomatic and self-limiting [51]. However, when migration results in root exposure or clinical symptoms, surgical removal may become necessary. This is supported by Póvoa et al. [39], who found that both root migration—even in the absence of exposure—and infection were among the most frequent postoperative issues, though their study reported a relatively low re-intervention rate of approximately 1.1%.

Less frequent indications comprised palpable roots (~2%), incomplete healing, and patient-requested extractions, each representing about 0.6% of cases. Additionally, isolated instances of periodontal disease, orthodontic treatment needs, soft tissue hyperplasia distal to the second molar, and intraoperative root displacement were noted, each occurring in fewer than 0.3% of cases [50]. Notably, in a substantial proportion of cases (*n* = 241), the rationale for reoperation was not specified.

#### 2.2.4. Reported Strengths and Limitations of the Procedure

Coronectomy offers a conservative surgical alternative to full third molar extraction, particularly in cases where the roots are in close proximity to the inferior alveolar nerve (IAN). Its primary strength lies in its ability to significantly reduce the risk of IAN injury, with nerve damage reported in less than 1% of cases [34,39]. The procedure is also associated with lower rates of alveolar osteitis [34,42] and less persistent postoperative pain compared to total extraction [44]. When performed in carefully selected patients—especially those with high-risk anatomical relationships—the technique provides a safe and effective method for managing impacted mandibular third molars [28,40].

Additionally, root migration, although observed in a varied percentage of cases, is generally a benign and self-limiting process [34,35,38,39]. Studies show that even when delayed extraction is required, there is no increased risk of IAN injury [46]. This reinforces the long-term safety profile of coronectomy in high-risk scenarios [41].

However, the procedure is not without limitations. The overall complication rate approaches 26%, with reoperation required in about 4.5% of cases, typically due to root exposure, infection, or pain [34]. Although most complications are minor, they emphasize the need for extended follow-up, as delayed issues may occur months or even years postoperatively [38,51].

Further limitations include the lack of standardized protocols for anatomical assessment, case selection, and follow-up. Key variables such as root morphology, distal space, and impaction depth are frequently underreported, despite their relevance in predicting complications like root migration or incomplete healing [42,46,51]. Imaging practices also vary significantly, with many studies relying solely on panoramic radiographs, which may inadequately assess IAN proximity. Cone-beam computed tomography (CBCT), although superior in high-risk cases, is not uniformly adopted [39,41,42]. Despite the highlighted advantages of the less invasive technique, the limitations described are not just technical but have direct implications for clinical practice, research and legal responsibility.

## 3. Discussion

### 3.1. Third Molar Extraction vs. Coronectomy: Indications, Procedure and Complications

Third molar surgery, like all surgical procedures, is not free from surgery-related complications. These complications may be intra-operative or post-operative (Table 7).

The most common complications related to wisdom tooth surgery are edema, trismus, soft tissue dehiscence due to improper flap management, while more severe but rarer ones include bone fractures, neurological injuries and conspicuous hemorrhage. Complications associated with the coronectomy procedure, on the other hand, depend on the necessity of reintervention at a second surgical time. This may, for example, be associated with migration of root remnants, with symptomatic retention of them, followed by postoperative site infection and pain. Considering that the initial surgical steps—namely, skeletonizing the mandible and exposing the root of the third molar—are similar for both coronectomy and extraction, it is to be expected that the reported incidence of lingual nerve injury (LNI) appears to be comparable between the two procedures. In detail, lingual nerve injury (LNI) is a less common complication overall but has also been observed at a lower rate following coronectomy. Peixoto et al. noted infact one case of temporary LNI in the extraction group, with no such incidents in the coronectomy cohort [28]. These data are consistent with the findings of larger systematic reviews, which estimate LNI incidence following coronectomy at approximately 0.1% [34,39]. This low incidence likely reflects the benefits of minimally invasive and atraumatic surgical techniques [49].

Conversely, inferior alveolar nerve injury (IANI) remains one of the most critical complications associated with mandibular third molar surgery. Numerous studies confirm that coronectomy significantly reduces the risk of IANI compared to full extraction. For instance, the results described by Peixoto et al. align with broader literature findings, which report IANI rates of approximately 0.76% for coronectomy [34], further corroborated by Póvoa et al., who documented a slightly lower rate of 0.59% [39]. This neuroprotective benefit is particularly evident in cases involving close proximity between the third molar roots and the inferior alveolar canal, as confirmed by studies using advanced imaging modalities [47,48].

Postoperative pain and trismus also appear to be more favorably managed in coronectomy. Broader evidence shows that pain affects approximately 10% of coronectomy patients [34], with comparable short-term discomfort to extraction but generally quicker resolution [36,52,53]. Williams and Tollervey also emphasized the role of reduced neural trauma in mitigating postoperative pain [44]. However, variability remains, as Póvoa et al. documented pain rates as high as 22%, suggesting that surgical technique, patient sensitivity, and follow-up duration can significantly influence outcomes [39].

Infections following coronectomy are relatively infrequent [28,34,39,45]. While slightly more frequent than in extraction procedures, these infections are generally minor and self-limiting. Notably, Yan et al. underscored the role of anatomical and periodontal factors in modulating infection risk, supporting a multifactorial interpretation of this complication [46].

Alveolar osteitis, or dry socket, is less commonly reported after coronectomy, likely due to root retention and preservation of the periodontal ligament. Peixoto et al. observed dry socket exclusively in the extraction group [28], and broader data place the incidence after coronectomy at approximately 1.2% [34], slightly lower than rates seen with full extraction. Póvoa et al. reported a nearly identical rate [39], while Hamad et al. found that dry socket occurred in only 0.5% of coronectomy cases compared to 3.7% in extractions [42]. This suggests a protective role of coronectomy in alveolar healing, attributed to the reduced disruption of local vascular and bone structures.

Additional complications remain rare. Lastly, healing and bone regeneration appear more favorable with coronectomy. Radiographic evaluations from both randomized controlled trials and observational studies demonstrate superior socket preservation and enhanced alveolar ridge maintenance in coronectomy cases, likely due to the minimal trauma involved and the maintenance of periodontal structures. Pang et al. and Bernabeu-Mira et al. have emphasized that retaining roots can help support bone healing, particularly distal to the second molar, contributing to more predictable long-term outcomes [38,54].

### 3.2. Lack of Procedural Standardization

The coronectomy procedure has been proposed by many authors as a solution to the risk of injury to the inferior alveolar nerve (IANI), especially when the roots of the third molar are in close proximity to the mandibular canal. Several randomized studies and systematic reviews have highlighted the advantages of this technique over traditional extraction methods, not only in terms of reducing the risk of IANI but also with regard to other clinical outcomes. Despite these findings, coronectomy remains a non-standardized procedure. This lack of standardization presents not only a clinical challenge for the operator performing the technique, particularly after an appropriate patient recruitment, but also a medico-legal concern. Medical, clinical, and surgical procedures must be defined within a framework of standardization, which includes evidence-based protocols or, in their absence, clinical practice guidelines or therapeutic recommendations. This framework is essential for reducing clinical variability among professionals, guiding clinicians to perform procedures correctly and ultimately improving the quality of care and patient safety.

#### 3.2.1. A Non-Standardized Approach

Despite a growing body of literature supporting coronectomy, its mini-invasiveness and advantages, the lack of standardized protocols for anatomical assessment, case selection and long-term follow-up continues to limit the consistency and generalizability of clinical outcomes. Critical variables such as root morphology, distal space, impaction depth, and third molar positioning are frequently underreported, although they play a pivotal role in predicting complications such as root migration, incomplete healing, post-operative pain or inferior alveolar nerve injury. The classification of impaction type and the use of risk stratification tools are inconsistently applied, further complicating clinical decision-making. Despite the demonstrated correlation between impaction characteristics and the likelihood of complications, many studies do not document key anatomical features like root curvature or the presence of adjacent second molars. This omission weakens the predictive value of preoperative assessments and may lead to suboptimal treatment choices. Similarly, demographic and clinical variables such as comorbidities and medication use are often omitted, which hampers the external validity of coronectomy findings. Although existing studies suggest that coronectomy is generally performed in young to middle-aged adults, the exclusion of patients with systemic conditions limits the applicability of results to broader populations.

In addition, post-operative imaging is not routinary performed. However, it may also be advisable to document the anatomical outcome of the procedure through postoperative radiographic evaluation, confirming successful crown removal and the apical positioning of the retained root fragments by at least 2–3 mm below the alveolar crest. With this aim, the same techniques proposed in the preoperative phases may be applied. In detail, Panoramic radiography remains the most commonly used imaging modality for assessing mandibular third molars [28,55,56], although its accuracy in determining proximity to the inferior alveolar nerve (IAN) is limited [41,54,57]. Studies have shown that panoramic imaging may misclassify high-risk cases, prompting recommendations for cone-beam computed tomography (CBCT) as the gold standard in complex cases [42,43].

CBCT offers superior three-dimensional visualization of root morphology, impaction depth, and IAN proximity, enabling more accurate risk assessment and surgical planning [46,48]. Its use has been shown to reduce unnecessary extractions and postoperative complications, including IAN injury and surgical site infections [38,45,51].

While other imaging techniques such as spiral tomography and periapical radiography are occasionally used, their limitations in spatial resolution make them less suitable for preoperative evaluation of impacted third molars [43,54,58]. Overall, CBCT plays a critical role in optimizing outcomes and should be integrated into the diagnostic workflow for high-risk third molar surgeries.

#### 3.2.2. Timing and Follow-Up

Furthermore, a significant limitation in the current understanding of coronectomy outcomes lies in the lack of standardized and adequately defined follow-up protocols. The heterogeneity in clinical monitoring practices and follow-up durations across studies hampers the ability to accurately assess the true incidence of late complications and the necessity for re-intervention. This gap in knowledge is particularly critical given that many complications—such as root migration, exposure, or postoperative infections—may emerge months or even years after the procedure. Bernabeu-Mira et al. [25], for instance, reported re-interventions occurring between 2 and 9 years postoperatively, underscoring the long-term nature of potential sequelae. Without uniform long-term surveillance, the rates of these events are likely underestimated. As highlighted by Kostares et al., the development and implementation of consistent follow-up protocols are essential for improving the reliability of outcome assessments and for guiding evidence-based clinical decision-making in coronectomy [32].

#### 3.2.3. Lack of Established Guidelines

Given these considerations, the use of coronectomy for the management of impacted mandibular third molars is not without potential medico-legal implications. To date practitioners are required to refer to recognized standards of good clinical practice. In light of this regulatory gap, when selecting a therapeutic approach for partially or fully impacted third molars, dental practitioners must rely on accepted standards of care. In this scenario, a complication or injury occurring during a coronectomy performed for an impacted third molar, may lead to difficulty in defending the clinical appropriateness of the procedure. In the absence of specific guidelines or consensus from the broader scientific community, the practitioner would be required to provide robust, high-quality scientific evidence to justify the technical validity of this surgical approach—evidence that, at present, remains limited and not widely endorsed. In addition, the absence of a stable perimeter of standardization of the procedure also places the clinician in the variability of choosing the technique and when to apply it, laying the groundwork for bridging medico-legal problems in case of postoperative complications, which do not have a well-defined follow-up period. The patient will also have to be well informed and the clinician will therefore in any case assume the risk of applying a procedure that to date has not been standardized.

#### 3.2.4. Proposals for Indications of Coronectomy

Coronectomy is primarily indicated in cases where imaging suggests a high risk of inferior alveolar nerve injury, such as when radiographic signs reveal root darkening, loss of lamina dura continuity, or displacement of the mandibular canal. These risk factors can be effectively identified through panoramic radiographs or, with greater accuracy, cone-beam computed tomography. Beyond nerve preservation, coronectomy may also be favored in patients with limited mouth opening or poor compliance, as it typically requires less operative time and is less invasive than full extraction. Nonetheless, the procedure is contraindicated in the presence of non-vital teeth, periapical pathology, extensive caries, or significant root mobility, as these conditions increase the risk of postoperative complications.

### 3.3. Informed Consent

#### 3.3.1. Possible Adverse Outcomes

Specifically, informed consent for coronectomy should include a clear explanation of potential nerve-related complications. Although the risk of lingual nerve injury (LNI) is relatively low and appears to be comparable between coronectomy and full extraction, patients should be made aware that this complication, albeit rare, may occur due to similar surgical access in both procedures. Conversely, coronectomy is associated with a significantly reduced risk of inferior alveolar nerve injury (IANI) compared to extraction. While IANI incidence in coronectomy is typically below 1%, extraction can present rates as high as 5%. This neuroprotective advantage is especially relevant in cases where the third molar is in close proximity to the inferior alveolar canal.

In addition to nerve-related complications, as with any surgical procedure, coronectomy carries the risk of other postoperative complications, though the overall incidence remains relatively low.

One of the more common issues is infection. These infections are generally mild and self-limiting but may require pharmacological treatment or, in rare cases, surgical follow-up. Although infection rates are slightly higher than those observed after complete extraction, the difference is not clinically significant. Nevertheless, factors such as the patient’s anatomical features, oral hygiene status, and underlying periodontal conditions may influence infection risk.

Another potential complication is alveolar osteitis, commonly known as dry socket. This condition is less frequently observed after coronectomy compared to lower third molar extraction, probably due to the preservation of the periodontal ligament and root structures, which promote a more stable healing environment.

Pulpal complications are exceedingly rare, with fewer than 0.1% of cases exhibiting symptoms related to the retained root segment. When the root remains well-embedded and unexposed to the oral cavity, it typically remains asymptomatic over the long term.

#### 3.3.2. Post-Operative Clinical Symptoms

Moreover, patients should be informed that pain and limited mouth opening (trismus) are common postoperative symptoms following third molar surgery, even if most discomfort is temporary and tends to resolve more rapidly than after extraction, likely due to reduced trauma to surrounding neural and soft tissue structures. However, evidence suggests that coronectomy is associated with lower pain intensity and faster functional recovery in the early postoperative period compared to full extraction. On average, postoperative pain affects approximately 10% of patients undergoing coronectomy, although reported rates vary and may reach up to 22% depending on individual factors such as surgical technique, tissue sensitivity, and follow-up duration.

#### 3.3.3. Two-Stage Surgery?

Patients should also be informed that while coronectomy generally presents a favorable safety profile, there remains a potential need for secondary surgical intervention. However, re-intervention is required in approximately 4–5% of cases. This does not imply that coronectomy merely constitutes a ‘two-stage extraction’, as most procedures do not progress to secondary surgery. When re-intervention is required, it is most commonly associated with root migration resulting in exposure or the onset of symptoms such as pain or infection, and is typically performed six months or more after the initial surgery, although delayed cases have been reported even several years postoperatively.

The most common indications for re-intervention include root exposure, postoperative infection, persistent pain, or residual enamel. Less frequently, patients may require additional surgery due to palpable root remnants, incomplete healing, or personal preference. Although root migration is a predictable outcome of coronectomy, it is generally benign and does not require treatment unless it leads to clinical complications.

Long-term follow-up is essential to monitor healing, detect late complications, and determine whether secondary intervention becomes necessary. The need for re-intervention may be influenced by patient-specific factors such as age, periodontal health, and anatomical risk features. Patients should be made aware that while most retained roots remain asymptomatic, a minority may require eventual removal.

#### 3.3.4. Intra-Operative Change in Procedure

It is also essential to inform the patient that in a small percentage of cases—ranging from approximately 3.5% to 3.6%—the coronectomy procedure may not be successfully completed as planned due to intraoperative complications such as root mobility, unfavorable anatomy, or technical difficulty. In such instances, the surgical approach may need to be modified intraoperatively, requiring immediate conversion to full tooth extraction. Patients should be made aware of this possibility and provide consent in advance for the surgeon to proceed accordingly if necessary.

Therefore prior to addressing the technical aspects of coronectomy as a surgical procedure, particular emphasis should be placed on issues concerning patient autonomy and the necessity for informed consent in dental interventions. According to the different and specific legislative frameworks, the failure to obtain truly informed consent could constitute an independent form of harm—namely, the violation of the patient’s right to self-determination—distinct from any damage caused by technical errors or physical injury resulting from treatment. A patient has the right not only to receive treatment (if they so choose) but also to be fully informed about the nature of the proposed therapeutic approach, its potential developments, and any viable alternative treatments.

It should be considered that informed consent provided by the patient is the fundamental legal and ethical basis for any medical or dental intervention. In the absence of such consent—except in situations involving mandatory treatment or medical emergencies—the act could be considered unlawful, even if performed in the patient’s interest. As a consequence, no medical treatment should be initiated or continued without the free and informed consent of the patient.

In this context, it is of paramount importance—ideally through written documentation—to provide the patient with a thorough, detailed, and intelligible explanation not only of the expected clinical outcomes of coronectomy but also of the short- and long-term complications and risks. These include the possibility of health deterioration following treatment, the potential failure of the procedure requiring subsequent intervention, the available therapeutic alternatives, and the necessity of close and prolonged follow-up. Patients must also be clearly informed that current scientific knowledge does not allow for reliable prediction of long-term outcomes following coronectomy. Furthermore, different surgical approaches for crown removal should be discussed in detail to ensure that the inherent risks of the chosen technique are shared and mutually understood.

The preventive role of informed consent turns out to be of pivotal relevance in light of coronectomy’s non-standardization and the potential medico-legal vulnerability.

### 3.4. Limitations of the Study

As this is a perspective article, the analysis reflects an expert but nonetheless subjective view of the authors, based, however, on the data that the most recent literature provides. The article does not include case law case histories, and in any case the medico-legal statistics could have differences and variability depending on the legal and ethical context of the state in which the law is applied.

## 4. Conclusions

Coronectomy has emerged as a safe and effective alternative to full extraction for managing high-risk mandibular third molars, particularly when there is close proximity to the inferior alveolar nerve. Many advantages have been pointed out by the many studies mentioned, including in terms of reduced postoperative complications compared with traditional technique. The procedure is associated with a significantly reduced incidence of inferior alveolar nerve injury compared to extraction, while lingual nerve injury is rare overall and occurs at comparable rates in both coronectomy and extraction, likely due to similar initial surgical approaches. In addition, patients undergoing coronectomy generally experience lower levels of postoperative pain and faster resolution of trismus, especially during the first week. Although pain rates vary among studies, the trend suggests that coronectomy results in less neural irritation and improved early recovery. Infections remain infrequent in both procedures, although slightly more common in coronectomy due to the presence of retained roots.

However, despite the usefulness of the technique to date, its application exposes the clinician to risks of both a clinical but especially a medico-legal nature. This is due to the technique’s current status as a non-standardized procedure with still uncertain long-term outcomes, variability in terms of imaging techniques, non-uniform surgical technique, as well as clearly defined criteria for re-interventions to enhance the reliability of clinical outcomes. Its use may potentially increase the practitioner’s medico-legal vulnerability, particularly in the absence of widely accepted clinical guidelines and consensus within the scientific community. The lack of studies with large numbers and the heterogeneity of management of even complications does not suggest a single line of decision making regarding this technique.

Moreover, clinicians should be able to demonstrate the presence of specific clinical indications, adherence to appropriate surgical timing and protocols, and the execution of the procedure with adequate technical proficiency.

Therefore the decision to perform a coronectomy also results in communication with the patient that must be absolutely clear and comprehensive about the risks and benefits and necessitates a thorough and well-documented informed consent process. Consequently informed consent must include not only the explanation of typical postoperative symptoms such as swelling, mild discomfort, and temporary reduction in mouth opening, but also the rare but potential complications. The importance of adherence to follow-up appointments and monitoring protocols should be emphasized to detect any delayed complications and ensure optimal healing.

Collectively, these issues highlight the need for further high-quality research and the development of standardized, guideline-based protocols to support the safe, effective, and legally defensible implementation of coronectomy in clinical practice. Standardization of the procedure would give the possibility to use this minimally invasive, conservative procedure related to lower risks of damage to nerve structures with greater safety on the part of the clinician and also towards the patient.

## Figures and Tables

**Table 1:** Root migration rate.

	Root Migration Rate
Di Spirito et al. [34]	12%
Hatano et al. [35]	85.29%
Dalle Carbonare et al. [36]	33%
Agbaje et al. [37]	40%

**Table 2:** Post-operative pain rate.

	Post-Operative Pain Rate
Hatano et al. [35]	18.63%
Póvoa et al. [39]	22.04%

**Table 3:** Infections rate.

	Infections Rate
Di Spirito et al. [34]	2.5%
Kostares et al. [45]	2.4%
Dalle Carbonare [36]	7.9%
Hatano et al. [35]	0.98%
Póvoa et al. [39]	3.95%

**Table 4:** Alveolar osteitis rate.

	Alveolar Osteitis Rate
Di Spirito et al. [34]	1.2%
Hamad et al. [42]	0.5%
Hatano et al. [35]	1.96%
Póvoa et al. [39]	1.1%

**Table 5:** Inferior alveolar nerve injury rate.

	IANI Rate
Di Spirito et al. [34]	0.76%
Dalle Carbonare [36]	0.5–1.3%
Hatano et al. [35]	0.98%
Póvoa et al. [39]	0.59%

**Table 6:** Re-intervention rate.

	Re-Intervention Rate
Di Spirito et al. [34]	4.5%
Dalle Carbonare [36]	0.5–1.3%
Hatano et al. [35]	4.90%
Póvoa et al. [39]	1.1%
Nowak et al. [51]	3.1%

**Table 7:** Intra-operative and post-operative complications of third molar surgery.

Intra-Operative Complications	Post-Operative Complications
Bleeding or Hemorrhage	Delayed Hemorrhage
Neurological injuries	Infections
Soft tissue lacerations	Trismus
Bone fractures	Edema
Injuries to contiguous teeth	Hematomas

## Data Availability

All data generated or analyzed during this study are included in this article.
